# From Bakelite to Biohazard: The Century-Long Rise of Microplastics

**DOI:** 10.7759/cureus.99627

**Published:** 2025-12-19

**Authors:** Joseph Mercola

**Affiliations:** 1 Independent Research, Midwestern University Chicago College of Osteopathic Medicine, Downers Grove, USA

**Keywords:** biodegradable polymers, environmental persistence, microplastic pollution, nanoplastic exposure, plastic production

## Abstract

The invention of Bakelite in 1907 marked the dawn of the synthetic polymer era, leading to exponential plastic production and widespread microplastic (MP) pollution. MPs (<5 mm) now permeate ecosystems and human tissues, and emerging evidence suggests that they may pose health risks ranging from reproductive to cardiovascular effects.

This narrative review aims to connect the historical growth of plastic production, the environmental spread of micro- and nanoplastics, evidence on human health effects, the development of candidate safer polymers, and the main policy responses. The specific objective is to identify key points for prevention, gaps in the evidence base, and priorities for future research and policy intervention.

A targeted literature search was conducted to identify studies on plastic production, micro- and nanoplastic pollution, human exposure and health effects, and policy responses. Literature was sourced from PubMed, Scopus, and Web of Science for articles published between January 1, 2010, and June 30, 2025, using combinations of the terms “microplastics,” “nanoplastics,” “plastic pollution,” “polymer production,” “human exposure,” “health effects,” and “policy responses.” Major reports from UNEP and WHO were also reviewed. Eligible were peer‑reviewed, English‑language articles reporting primary environmental or human health data, quantitative syntheses, or policy analysis relevant to human exposure/outcomes. We excluded non‑human studies without clear human relevance, conference abstracts, non‑English publications, and non‑seminal work before 2010. Screening yielded 250 records; 162 met criteria for narrative synthesis. Due to heterogeneity in designs, exposure metrics, and outcomes, no risk-of-bias assessment or meta-analysis was conducted. Quantitative findings are summarized descriptively with effect sizes and confidence intervals when available.

Global plastic production increased from about 2 million metric tons in 1950 to more than 450 million metric tons by 2018, while only 9-20% of plastic waste underwent recycling. Most waste entered landfills, incinerators, or the environment. Mismanaged waste adds an estimated 4.8 to 12.7 million metric tons of plastic to the oceans each year, where larger items fragment into MPs and nanoplastics that persist, accumulate across food webs, and carry co-pollutants. Human studies now detect these particles in blood, placenta, lung tissue, and atherosclerotic plaques, and one cohort reported higher rates of myocardial infarction, stroke, or death among patients whose carotid plaques contained MPs or nanoplastics. Additional evidence links exposure with endocrine disruption and reduced sperm quality, yet effect sizes vary, and most data remain observational, which underscores the need for longitudinal and mechanistic research that defines causal pathways from particle characteristics and dosimetry to specific health outcomes. The available evidence indicates that historical decisions about polymer design and plastic production now drive widespread micro- and nanoplastic exposure with plausible cardiovascular, endocrine, and reproductive consequences, although causal pathways remain incompletely defined. Coordinated action that aligns safer polymer design, exposure reduction, longitudinal health research, and binding international policy will be necessary to curb micro- and nanoplastic contamination and to protect human health.

## Introduction and background

The invention of Bakelite in 1907 heralded the modern plastics era [1]. As the first synthetic phenolic plastic, Bakelite entered production as an electrical insulator and durable household material, launching the era of synthetic polymers [2]. Its success spurred the development of a wide range of plastics, including polyethylene, polypropylene, and polyvinyl chloride, that offered unprecedented malleability, strength, and chemical resistance [3]. Over the ensuing century, synthetic polymers revolutionized industry and daily life due to their durability, moldability, and low cost [4]. By the mid-20th century, plastics had displaced traditional materials across multiple sectors, from packaging and textiles to automotive and medical devices [5].

What was once celebrated for its durability is now recognized as a persistent pollutant [6]. Jambeck et al. estimated that 4.8-12.7 million metric tons of plastic debris enter the oceans annually from coastal mismanagement alone [7]. The cumulative effect of this leakage has created what researchers describe as a global "plastic smog" - a diffuse load of microplastic (MP) fragments pervading surface waters and sediments [8]. Most synthetic plastics resist biodegradation and do not mineralize; instead, they can persist for decades or centuries in the environment [9].

In this way, the triumph of polymer science has become tightly linked to one of its most urgent legacies: the global burden of MPs now confronting ecosystems and public health alike [10].

We now face a rapidly expanding evidence base on MPs and nanoplastics (NPs) yet have a fragmented view of risk. Existing reviews often treat plastic production trends [11,12], environmental fate, toxicology, and policy responses as separate topics. These reviews rarely integrate the historical rise of synthetic polymers with contemporary exposure patterns and human health outcomes, or with the policy responses that aim to mitigate those risks.

This narrative review addresses that gap. It focuses on four linked domains: the evolution of synthetic polymers and MP generation, environmental distribution and exposure pathways, evidence for human health effects, and the policy and regulatory measures that respond to this emerging burden. The primary aim is to synthesize what we know about how the history and design of synthetic polymers shape current MP exposure and health risk, and how policy efforts align, or fail to align, with that knowledge. Established associations and hypothesized causal pathways are clearly distinguished, and priority directions for longitudinal and mechanistic research are highlighted.

Key concepts include MPs (plastic fragments <5 mm, often from larger plastic degradation [6]) and NPs (<1 μm, more bioavailable due to cellular uptake [10]).

## Review

Methods

Literature was sourced from PubMed, Scopus, and Web of Science (2010-2025) using the keywords "microplastics," "plastic pollution," "polymer production," "health impacts," and "policy responses." Inclusion criteria were peer-reviewed English-language articles with primary data, meta-analyses, or policy reviews; exclusion criteria were non-human studies without health relevance or pre-2010 non-seminal works. Approximately 250 sources were screened, with 156 retained. Risk of bias was informally assessed via journal impact factor and citation consensus; no formal tool was used, given the narrative scope. Quantitative data were summarized descriptively due to heterogeneity; no meta-analysis was performed, but effect sizes from key studies are reported with CIs where available.

Historical surge in polymer output - key milestones

Global plastic production climbed from just 2 million metric tons in 1950 to more than 450 million metric tons by 2018, making plastics one of the fastest-growing material classes in modern history (Table 1). Cumulatively, ~8.3 billion tons of plastic had been manufactured worldwide [12,13].

**Table 1:** Key milestones in global plastic production The quasi-exponential rise correlates with a 10-fold increase in ocean plastic leakage, underscoring the need for production caps as advocated in recent global treaties.

Year	Production (Million Tons)	Key Milestone/Source
1950	2	Post‑WWII boom begins [6]
1990	100	Thermoplastics dominate [7]
2018	450	Cumulative production: 8.3 billion tons [8]
2024	500+	Production continues to rise; ocean leakage: 8.8 million tons/year; industry projections

**Figure 1:**
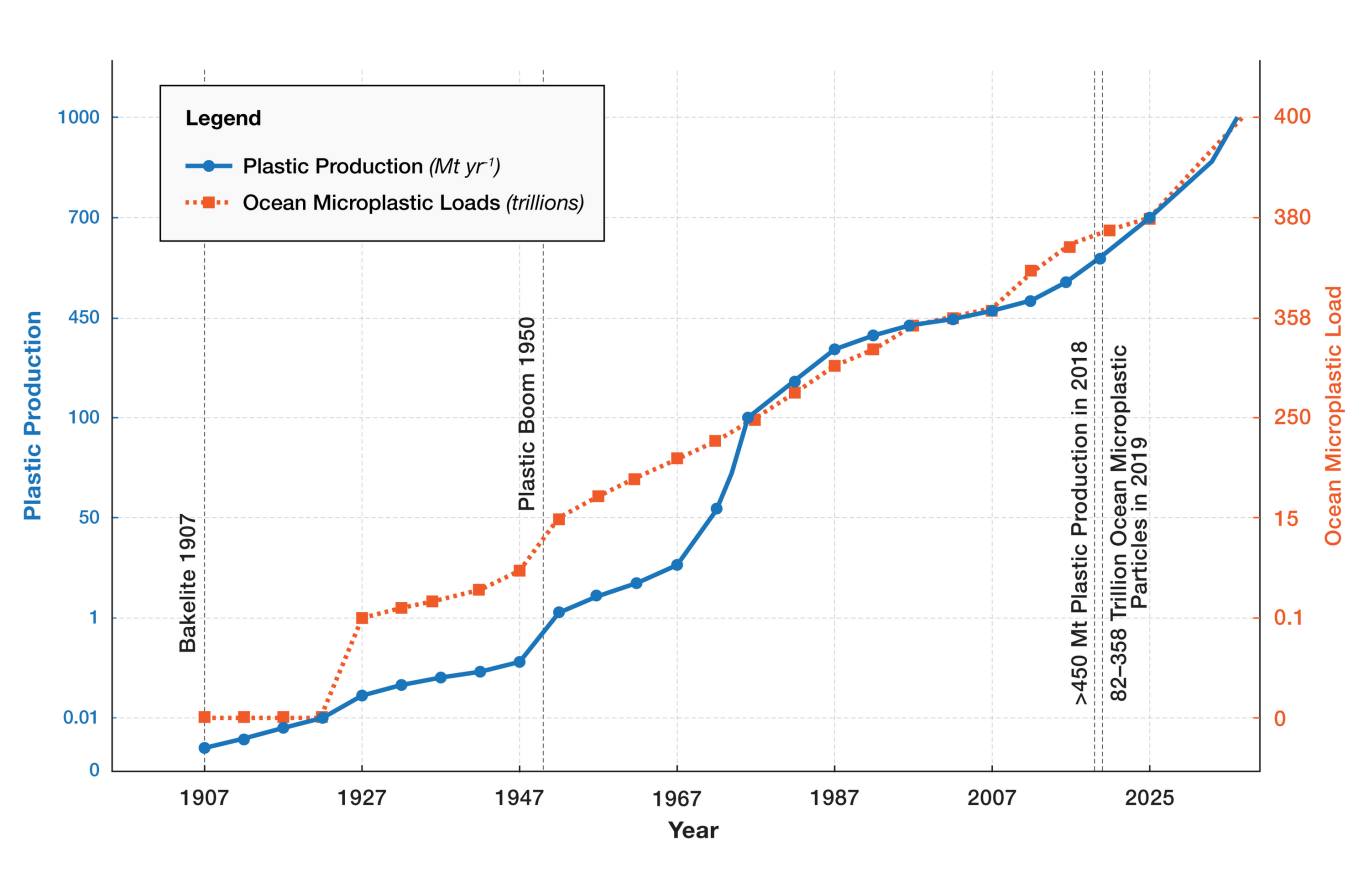
Global plastic output and surface-ocean MP burden Global plastic output and surface-ocean MP burden have risen quasi-exponentially since 1950, illustrating a tightly coupled anthropogenic supply-pollution dynamic. MP: microplastic.

These production trends translate into MP burden because every ton of durable polymer adds to a growing in-use and waste reservoir that can fragment into MPs and NPs rather than disappear. The quasi-exponential rise correlates with a 10-fold increase in ocean plastic leakage [7], underscoring the need for production caps as advocated in recent global treaties [14]. These figures put into perspective the genesis of the MP era - a direct result of historical plastic "overshoot" and waste mismanagement that has allowed plastics to leak into oceans, rivers, and soils (Figure 1) [15]. Critically, the lag between production increases and environmental detection reflects both accumulation dynamics and evolving analytical capabilities. MPs detected in remote environments today represent decades of prior emissions, making current interventions insufficient to address legacy contamination even if production ceased immediately. This temporal disconnect between cause and effect underscores why production-focused policies must be implemented urgently - environmental MP burdens will continue rising for decades even under optimistic intervention scenarios.

Economic and industrial drivers of plastic proliferation

One key driver of plastic proliferation is the rise of single-use and disposable products [16]. It is estimated that as of 2020, about 40% of plastic produced annually was for packaging and other short-lived items [17]. Such items become waste within months, providing a continuous stream of plastic waste [18]. The petrochemical industry's pivot in recent years toward plastics has further amplified production capacity [19]. Projections by industrial and governmental analyses forecast that if business-as-usual continues, global plastic production could triple by 2060. This would mean roughly 1.2 billion tons produced per year, a scenario virtually guaranteeing magnified MP pollution unless mitigated [20].

Historical polymer output and waste management practices set the scale and trajectory of today’s MP burden, and environmental and human exposure records mirror these production curves.

By the 2010s, MPs were being detected in even the most remote environments [21]. For instance, glacier cores in the European Alps and Arctic sea ice in the 2010s contain embedded plastic fibers and fragments, reflecting deposition from increased atmospheric and oceanic burdens [22]. Likewise, deep-sea sediment cores show a steep rise in plastic particles starting mid-century, mirroring the sharp production increase in the post-1950 period [23].

In human dietary exposure, trends also follow production: studies note that MP contamination in seafood and sea salt has climbed in tandem with ocean MP counts, which roughly doubled between 2000 and 2020 in some regions [24]. There’s also a temporal correlation with rising human tissue burdens of MPs [10]. The ubiquity of "plastic age" markers in our bodies - from chemical plasticizers in urine to MPs in blood - represents an observational correlation with living amidst the highest polymer production volumes in history [25].

Weathering mechanisms driving plastic fragmentation into micro- and nanoparticles

In the environment, macroplastic debris is subject to a suite of weathering processes that progressively break it into MP (≤5 mm) and NP (<1 µm) particles that infiltrate water, soil, and air compartments [26].

Chemical Degradation

Photodegradation is a primary driver: solar ultraviolet (UV) radiation oxidizes polymer chains, making them brittle [27]. UV exposure triggers chain scission and carbonyl formation, increasing hydrophilicity and fragmentation [28]. Over weeks to months of sun exposure, plastics develop surface cracks and discoloration. The weakened plastic then fragments under mechanical stress [29]. Thermal oxidation similarly embrittles polymers through repetitive heating/cooling cycles in sunlight [29]. The result is a gradual reduction of plastic items into millimeter- and micrometer-scale pieces [30]. Notably, UV exposure also produces micro- and nano-scale weathering byproducts such as dissolved organic carbon and methane from certain plastics, but the solid fragment generation is of most concern ecologically [31].

Physical Fragmentation

Mechanical abrasion further accelerates fragmentation [6]. In marine environments, waves and sand scour large debris, chipping off micro-sized particles [32]. Beach sands act as sandpaper on washed-up plastics, and river rocks abrade plastic litter in waterways [33]. Fishing gear and plastic ropes, for example, release microfibers due to constant friction and tension [34]. Similarly, vehicle tire wear on roads produces abundant synthetic rubber MPs via abrasion [35].

Biological Breakdown

Additionally, the action of biota contributes: some marine organisms bore into plastic (e.g., crustaceans scraping algae off plastic surfaces), creating microfragments [36]. Microbes can colonize plastic surfaces forming a plastisphere, and while true biodegradation into biomass/CO_2_ is minimal for most polymers, biofilm growth can weaken plastics and promote fragmentation [37].

Importantly, the generation of NPs is an inevitable continuation of these processes [38]. As MPs break down further, nanometer-scale fragments emerge, though detecting them is analytically challenging [39]. Laboratory experiments confirm that prolonged UV exposure and mechanical milling of common plastics yield nanoscale particles in the 100-1000 nm range [40]. Given enough time, a single plastic item could theoretically produce billions of NP particles [41]. Environmental sampling indeed has identified NPs in ocean water and polar ice cores, though quantifying their abundance remains difficult [42].

Overall, weathering via UV, thermal cycling, mechanical forces, and limited biodegradation synergistically converts durable plastics into ever-smaller debris. This continuous fragmentation ensures that, absent effective waste capture, the MP and NP pool will only grow over time [40].

These weathering processes directly determine human and ecological exposure routes. UV-fragmented MPs in surface waters enter marine food webs through filter-feeding organisms such as mussels and oysters, bioaccumulating up trophic levels to fish consumed by humans. Mechanically abraded tire particles wash into stormwater systems and ultimately rivers and coastal zones, where they settle in sediments or remain suspended in the water column.

**Figure 2:**
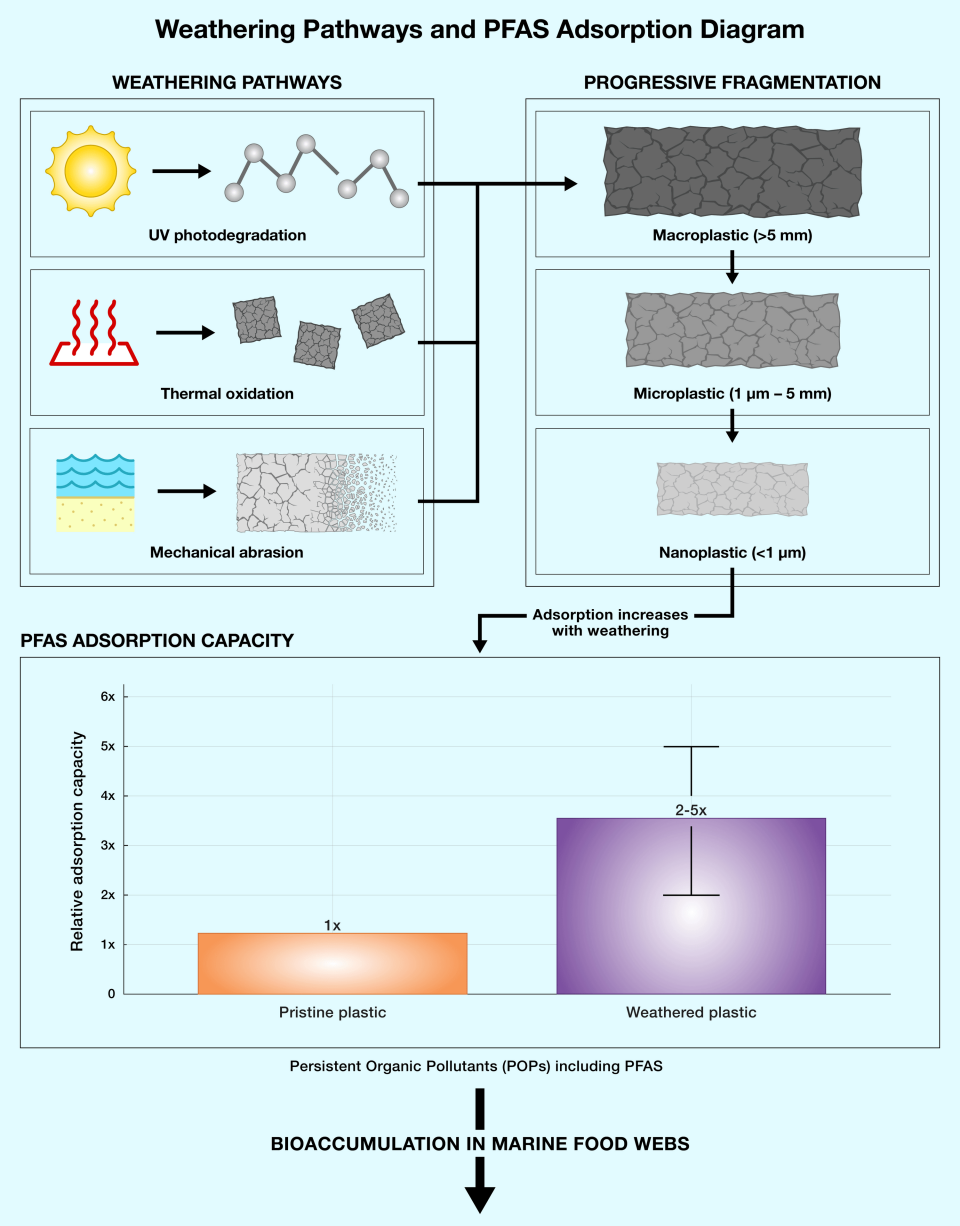
Weathering pathways and PFAS adsorption capacity This figure shows how UV photodegradation, thermal oxidation, and mechanical abrasion progressively fragment macroplastics into microplastics and nanoplastics. Weathered particles demonstrate 2-5x higher adsorption capacity for POPs such as PFAS compared to pristine plastics, amplifying bioaccumulation risks in marine food webs. This figure is adapted from Sait et al., 2021 [28]. POPs: persistent organic pollutants; PFAS: per- and polyfluoroalkyl substances.

Atmospheric transport of weathered fragments enables long-range deposition even in pristine environments, explaining MP presence in Arctic snow and remote mountain lakes far from population centers. Critically, weathering also exacerbates bioaccumulation potential and chemical toxicity. Weathered MP surfaces show 2-5 times higher adsorption capacity for persistent organic pollutants (POPs) such as per- and polyfluoroalkyl substances (PFAS) compared to pristine plastics (Figure 2) [28], amplifying chemical exposure when contaminated particles are ingested by biota or humans. This "Trojan horse" effect means that weathered MPs serve not only as physical contaminants but as vectors for concentrated chemical delivery into biological systems.

Quantitative assessment of MP loads in global ecosystems as of 2025

Today, MPs and NPs are recognized as pervasive contaminants spanning remote and urban environments [43]. They have been found from the deepest marine trenches to the summit of Mount Everest. More than 1,300 wildlife species, from plankton and shellfish to birds and whales, are known to ingest or become entangled in plastic fragments [44]. Such encounters cause physical harm, such as gastrointestinal blockage, entanglement injuries, and can be fatal, contributing to biodiversity loss [13].

Over the past decade, researchers have attempted to gauge the scale of MP contamination in major ecosystems [45]. In the world's oceans, extensive surface trawl surveys and modeling provide the most robust estimates [46]. A 2023 meta-analysis of 11,777 ocean stations estimated that 82-358 trillion plastic particles, mostly MPs, were afloat in the surface layer of oceans as of 2019 [47]. This corresponds to a floating mass of 1.1-4.9 million tons of small plastics globally. The study reported an accelerating accumulation since 2005, reflecting continued inputs [46]. Importantly, these figures exclude the even greater loads of MP likely present in deep-sea sediments and the water column below the surface [48]. Seafloor sediment cores from remote ocean basins reveal MP deposition rates doubling roughly every 15 years since the 1940s, mirroring plastic production trends [49]. One recent estimate put the amount of MP on the ocean floor at 14 million tons, based on extrapolation from limited deep-sea samples [50].

Terrestrial ecosystems are similarly inundated with plastic debris. Soils near population centers contain accumulated MPs from decades of litter, wastewater sludge application, and atmospheric fallout [56]. Agricultural soils are a particular hotspot due to the use of plastic mulches and irrigation materials that degrade in situ [57]. Global surveys of agricultural soils have found MP concentrations ranging from thousands to hundreds of thousands of particles per kilogram of dry soil [58]. Riverine and estuarine sediments downstream of cities often show MP levels in the hundreds of particles per kilogram of sediment [59]. An emerging concern is the atmospheric transport of MPs: fine plastic particles become airborne via wind erosion of land and sea surfaces. Atmospheric models estimate that tens of thousands of tons of MP fibers and fragments are deposited from the atmosphere onto land and oceans each year [60]. Even remote alpine and polar regions receive plastic deposition via long-range air currents, evidenced by MPs found in Arctic snow and high-altitude mountain lakes [61].

Freshwater systems also provide a conduit and repository for plastics [62]. The Great Lakes, for example, are estimated to contain billions of plastic particles, and river surface concentrations downstream of cities can exceed 100,000 MP particles per cubic meter [63]. No ecosystem has been spared: from urban air and city tap water to polar sea ice and abyssal sediments, MP pollution is now an entrenched component of the global geochemical cycle (Table 1) [64-66]. Current best estimates put the total environmental MP burden on the order of tens of millions of tons, and under business-as-usual plastic use, annual emissions of MPs to the environment are projected to double by 2040 [67]. These figures underscore the challenge ahead in monitoring and mitigating a pollutant that has, in a few decades, achieved planetary ubiquity [68].

The ecosystem burden estimates presented in Tables 2, 3 derive from different sampling methodologies and should be compared cautiously. Ocean surface estimates rely on standardized trawl protocols developed through international coordination, while soil and atmospheric data employ gravimetric or spectroscopic methods with variable detection limits. Deep-sea sediment mass estimates are extrapolated from limited core samples and carry substantial uncertainty. Where multiple estimates exist for the same compartment, ranges reflecting methodological variation are presented rather than selecting single values. Priority was given to studies with larger sample sizes, broader geographic coverage, and peer-reviewed methodology validation.

**Table 2:** Key quantitative metrics in microplastic research Quantitative estimates were derived from peer-reviewed meta-analyses and primary studies. Extrapolations (where noted) are based on linear growth models validated against 2020-2024 data [15]. Ocean surface particle estimates reflect surface trawl surveys with mesh sizes typically ≥300 μm, systematically underestimating smaller MP and NP fractions. Tire wear emission ranges reflect variability in driving conditions, vehicle weight, and measurement methodology. N/A: not available or not reported in source literature; CI: confidence interval; MP, microplastic.

Metric	Value	95% CI/Range	Source
Annual Ocean Leakage (2015)	8.8 million tons	4.8-12.7	Jambeck et al., 2015 [7]
Cumulative Global Plastic Production (1950-2018)	8.3 billion tons	N/A	Biyani et al., 2025 [15]
Global Recycling Rate	9-20%	N/A	UNEP, 2024 [51]
Ocean Surface MP Particles	82-358 trillion	N/A	Eriksen et al., 2023 [46]
Deep‑Sea Sediment MP Mass	~14 million tons	Estimate extrapolated	Barrett et al., 2020 [52]
Human Lung Tissue MP	11 particles/g tissue	N/A	Street et al., 2025 [5]
Seafood MP Ingestion (Europe)	~11,000 particles/year/consumer	N/A	Van Cauwenberghe and Janssen, 2014 [53]
Tire Wear MP Emission	4 mg/km/tire	Range: 3-6	Saladin et al., 2024 [54]
Microfiber Shedding (polyester garment)	~0.5 g/garment lifetime	N/A	Liu et al., 2022 [55]

**Table 3:** Global microplastic burden by ecosystem (2025 assessment) Some compartments lack either particle concentration or mass estimates due to current limitations in sampling and extrapolation methods. For deep-sea sediments, mass estimates are available but particle concentrations are not consistently reported. For agricultural soils, atmospheric compartments, and freshwater systems, localized concentration data exist, but comprehensive global mass estimates are unavailable or highly uncertain. trn: trillion; t yr⁻¹: tons per year.

Ecosystem	Median Particle Concentration (Units)	Mass Estimate (Mt)	Temporal Trend	Key Reference
Oceans	82-358 trn particles	1.1-4.9	Increasing since 2005	Eriksen et al. (2023) [46]
Deep-sea sediment	–	14	Doubling deposition every 15 years	–
Agricultural soil	10³-10⁵ particles kg⁻¹	–	Accumulating over decades	Nizzetto et al. (2016) [69]
Atmosphere	≈10⁴ t yr⁻¹ deposition	–	Global dispersal increasing	–
Freshwater	≤10⁵ particles m⁻³	–	Widespread and persistent	–

Human exposure and potential health effects

A central question is whether ubiquitous MP and NP exposure translates into tangible health risks. Plastics once carried a reputation as biologically inert materials, yet modern work suggests that cells do not simply ignore them [70]. Many cells have been shown capable of internalizing plastic particles of roughly 50-100 nm through endocytosis and engulfing larger particles through phagocytosis, while ultrafine particles below about 50 nm may have the ability to cross cell membranes directly.

Particles in the MP and NP size range cross epithelial barriers in the gut and lungs, enter the bloodstream, and appear to accumulate in internal organs [71]. Biomonitoring studies have already detected plastic particles in most sampled human blood specimens [25], and tissue analyses now report particles in human placenta [72], lung tissue [73], and atherosclerotic plaques [74]. Recent work also reports NPs within human brain tissue, which implies passage across the blood-brain barrier and raises concern about direct neurotoxic effects [75,76].

Laboratory and animal studies outline plausible toxicological pathways. Ingested MPs can alter gut microbial communities and provoke local intestinal inflammation [77]. At the cellular level, MP and NP exposure can generate ROS, cause oxidative stress, and damage mitochondria, with reductions in ATP production and evidence of broader cellular injury [78]. Reviews of this literature now suspect adverse effects across multiple organ systems, with the digestive, respiratory, and reproductive systems of particular concern [10].

Evidence also begins to connect internalized plastics with specific clinical outcomes. In cardiovascular research, investigators have detected MPs and NPs in carotid atherosclerotic plaques and reported higher rates of myocardial infarction, stroke, or death among patients whose plaques contained these particles compared with those whose plaques did not [74].

Emerging human studies also link MP exposure to increased risks of preterm births and inflammatory bowel disease, and NPs to neuroinflammation and cognitive impairments [79], which supports concern about potential effects on the central nervous system once particles reach the brain.

Reproductive toxicity provides another key signal. Animal models show that plastic particles can accumulate in ovaries and testes, impair gametogenesis, disrupt sex hormone levels, and reduce fertility [80]. Human data, although still limited, now associate MP exposure with endocrine disruption and reduced sperm quality [81,82]. Beyond the particles themselves, leached plastic additives such as bisphenols and phthalates act as endocrine disruptors, mimic endogenous hormones, and interfere with thyroid and reproductive axes [83]. Together, this mix of particle effects and chemical co-exposures points to a credible risk for reproductive and developmental health.

Despite these advances, major knowledge gaps remain. Effect sizes across studies vary, most human data remain observational, and clear dose-response relationships are still uncertain. Key questions include how particles move across biological barriers, how long they persist in specific tissues, and which combinations of size, shape, polymer type, and additive load pose the greatest risk. Addressing these gaps requires longitudinal and mechanistic research that links particle characteristics and internal dosimetry to defined health outcomes, rather than reliance on cross-sectional associations alone.

Innovations in green polymer chemistry to reduce long-term ecotoxicological risk

Mitigating the MP crisis calls not only for managing existing waste but also for reimagining the materials we use [84]. In recent years, green polymer chemistry innovations have focused on designing plastics that minimize environmental persistence and toxicity [85]. One avenue is the development of truly biodegradable polymers that can fully break down into benign substances in natural environments. For example, polyhydroxyalkanoates are bio-based polyesters synthesized by microbes; they can biodegrade in soil and marine settings within months, ultimately converting to CO₂, water, and biomass [86]. Polylactic acid (PLA), derived from corn starch or sugarcane, is another widely used bioplastic that degrades under industrial composting conditions [87].

Challenges and scalability issues

However, PLA requires high temperatures and specific conditions to decompose efficiently, and in cool marine waters, it may persist for years [87]. Research is ongoing to tweak PLA formulations to enhance their breakdown in ambient conditions [88].

Another strategy is embedding depolymerization triggers into plastics [89]. Chemists are exploring polymers with cleavable bonds that can be broken down on demand [90]. For instance, adding UV-sensitive linkages that fragment when exposed to certain wavelengths could help plastics self-degrade after a programmed lifespan [91]. Similarly, incorporating biodegradable segments or weak links in polymer chains can accelerate fragmentation and biodegradation [89]. A related innovation is enzyme-enabled plastics: integrating enzymes or enzyme-mimicking catalysts into plastics that remain inactive during use but can be activated to depolymerize the material at the end of life [92]. One celebrated breakthrough involved engineering a variant of PETase, a bacterial enzyme that can digest PET plastic [93]. Researchers improved its activity and combined it with another enzyme to create a cocktail that can break down a PET bottle in days [94]. While still far from commercial deployment, such advances raise the possibility of plastics that can be rapidly "composted" or recycled enzymatically, preventing long-term MP generation [95].

Green chemistry also targets the toxic additives in plastics. There is movement toward safer plasticizers and flame retardants that do not have endocrine activity. For example, bio-based plasticizers from citric acid or castor oil are being developed to replace phthalates in flexible PVC [96]. Non-halogenated flame retardants can substitute for brominated compounds to reduce persistence and bioaccumulation. The concept of benign-by-design plastics pushes manufacturers to consider the end-of-life and ecotoxicity profile during the polymer design stage, not after. This includes ensuring any breakdown products are non-toxic. For instance, polymer chemists have synthesized polyurethane foams using glycolates that degrade into soluble, non-toxic monomers in seawater, addressing the issue of lost fishing gear [97].

Biopolymers made from waste or renewable feedstocks also show promise in reducing long-term impact [98]. "AirCarbon," a PHA-type polymer made by feeding captured methane or CO₂ to bacteria, yields a plastic that is carbon-negative in production and biodegradable after use [99]. However, while innovations like PHA bioplastics show promise [100], scalability remains limited by high production costs (2-5x conventional plastics) and land-use competition with food crops. Algae-based and cellulose-based polymers are emerging as well, which may break down more readily in marine environments [101]. Yet, scaling these alternatives presents challenges in cost, performance, and resource use, such as farmland for biopolymer crops vs. food [102].

It is important to note that not all so-called "biodegradable" plastics are environmentally benign. Some marketed as "oxo-degradable" simply have additives that cause the plastic to fragment faster into MPs, without true biodegradation -- a practice now banned in the EU due to MP and NP pollution concerns [103]. Thus, rigorous standards and certifications for biodegradability in natural conditions (marine, freshwater, soil) are needed. Moreover, life-cycle assessments (LCAs) are crucial: a polymer that biodegrades but has a high carbon footprint or other pollution upstream might solve one problem while worsening another [104].

LCA of MPs: From cradle to grave (and back again)

An LCA lens shows that MPs arise at every stage of a plastic product’s life, from polymer production to waste management and environmental recirculation [105-107]. This perspective clarifies that MP pollution does not begin at “end-of-pipe,” but reflects cumulative decisions about how plastics are designed, produced, used, and discarded across decades of rising polymer output.

Production and Manufacturing

The life cycle begins with polymer production, typically from fossil fuels [108]. Even before products reach consumers, primary MPs enter the environment through pre-production pellet (nurdle) losses and industrial dust. Nurdles, which measure up to about 5 mm, spill during transport and handling; global losses may reach 230,000 tons per year, and these pellets later fragment into smaller MPs and NPs [109-111].

Additional releases occur in the form of fine polymer powders and dusts near production facilities [112].

Usage Phase

During use, abrasion and wear convert macroplastics into MPs continuously. Synthetic textiles shed microfibers during washing; a single polyester garment may release on the order of 0.5 g of fibers over its lifetime, and conventional wastewater treatment captures only part of this load [55,113]. Tire wear is another major diffuse source: each tire can lose roughly 1.5 kg of rubber-plastic tread, much of it as particles in the PM₁₀ and PM₂․₅ size ranges that accumulate as road dust or wash into waterways [114]. Everyday actions such as scratching plastic surfaces or opening and closing caps add smaller but persistent inputs [113]. Because these releases occur during normal use, they often bypass downstream controls.

End-of-Life Waste Management

End-of-life handling strongly shapes the scale and timing of MP generation [115]. In landfills, plastics weather slowly, and leachate can carry MPs into surrounding soils and groundwater [115]. Open dumps in low- and middle-income regions allow debris to escape directly into terrestrial and aquatic systems, where it fragments further [116]. Incineration can reduce plastic mass and limit MP formation when facilities operate with high combustion efficiency, but residual particulates and air pollution control waste may still contain polymer fragments. Mechanical recycling lowers demand for virgin plastic, yet generates plastic dust and fibers within facilities, with elevated occupational exposure documented among recycling workers [117]. Thus, waste management practices determine whether the large stock of discarded plastic becomes a long-term MP source or is at least partially contained.

Environmental Circulation and Feedback Loops

Once released, MPs circulate among air, water, sediments, and biota, and re-enter human exposure pathways [118]. Marine and freshwater organisms ingest MPs, which then move through food webs. Shellfish, for example, can accumulate MPs that later appear in human diets; European consumers may ingest on the order of 11,000 MP particles per year from shellfish alone [119-121]. Atmospheric transport and deposition deliver MPs to remote regions and to indoor and outdoor air, adding inhalation pathways to dietary intake. From an LCA perspective, these feedback loops mean that plastic “waste” continues to generate exposures long after production and consumption events have occurred.

Taken together, the life-cycle view highlights key leverage points for mitigation. Upstream interventions, such as reducing short-lived applications, redesigning products and packaging to shed fewer particles, and substituting less friable materials, prevent MP generation at the source [67,122]. Midstream measures, including washing machine microfiber filters or emerging tire particle capture technologies, can limit releases during use [113,123]. Downstream improvements in collection, landfill design, recycling, and spill prevention reduce secondary MP formation from mismanaged waste [124,125]. Scenario analyses suggest that broad adoption of best-practice waste management could reduce plastic leakage to the oceans by roughly one-third by 2040, even without major changes in production, underscoring the importance, but also the limits, of end-of-pipe controls in the context of ongoing production growth [126].

Quantitative Life-Cycle Comparison

A comprehensive LCA underscores one more point: substitution and trade-offs [127]. Alternatives to plastics may have other environmental costs [128]. The goal from a life-cycle perspective is an optimized system where essential uses of plastic are managed in closed loops, non-essential uses are phased out, and materials are selected to minimize fragment generation [117]. Importantly, LCA teaches that MPs are an externality not priced into plastics' life cycle [129]. Incorporating the cost of pollution (e.g., via extended producer responsibility or plastic taxes) could incentivize industry to design plastics that are less prone to fragmentation or easier to recapture at the end of life [130].

To provide concrete comparative data, Table 4 summarizes key life-cycle metrics across different plastic types. This quantitative comparison demonstrates that while bioplastics offer advantages in MP leaching risk and biodegradation, they are not universally superior across all environmental metrics. Recycled PET achieves significant carbon footprint reduction (70-80%) compared to virgin material while maintaining comparable performance, though MP fragmentation risk remains elevated [28]. PHA bioplastics show the most favorable profile for MP risk mitigation but face economic scalability challenges [100]. These trade-offs underscore the need for context-specific material selection rather than blanket substitution policies.

**Table 4:** Life‑cycle assessment comparison of plastic types Production costs for bioplastics remain 2-5x higher than conventional plastics, limiting scalability. Marine biodegradation times represent optimal conditions; actual degradation may be slower depending on temperature and microbial community composition. CO₂e: carbon dioxide equivalent; MP: microplastic.

Plastic Type	CO₂e/kg Production	MP Leaching Risk	Biodegradation Time	Source
Virgin PET	3-4 kg CO₂e	High	>100 years	UNEP, 2024 [51]
Recycled PET	0.5-1.2 kg CO₂e	Medium (20% reduction)	>100 years	Sait et al., 2021 [28]
PHA Bioplastic	0.5-1.2 kg CO₂e	Low	6-12 months (marine)	Yeo et al., 2024 [100]
Polylactic Acid (PLA)	0.8-1.5 kg CO₂e	Medium	6-24 months (industrial compost); years (marine)	Royer et al., 2023 [101]

Policy instruments targeting upstream plastic waste and additive use

Addressing MP pollution at the source requires comprehensive policy interventions targeting plastic production, use, and waste management (Table 5) [131]. One set of instruments focuses on reducing upstream plastic waste generation [132]. Many jurisdictions have introduced bans or levies on single-use plastic items that frequently become litter. Dozens of countries now ban lightweight plastic shopping bags or impose fees to discourage their use [133]. The European Union's Single-Use Plastics Directive (2019) bans items like straws, cutlery, and cotton swabs made of plastic, and mandates reduced consumption of plastic food containers [134]. These measures aim to cut off major contributors to plastic leakage into the environment. Similarly, several nations and states have banned oxo-degradable plastics (which break into MP) to prevent false solutions [131].

**Table 5:** Comprehensive policy instrument descriptions Policy momentum is shifting, with bans, levies, and global treaties that collectively aim to curtail intentional MP emissions. MPs: microplastics.

Instrument	Jurisdiction/Year	Scope	Anticipated MP Reduction
Microbead‑Free Waters Act [143]	US/2015	Bans manufacture and sale of rinse‑off cosmetics containing plastic microbeads. Applies to products like facial scrubs and toothpastes.	Eliminated a significant point‑source of MPs. Quantitative reduction not specified.
EU Single‑Use Plastics Directive [161]	EU/2019	Bans plastic items such as straws, cutlery, cotton swabs, and certain polystyrene containers. Mandates consumption reduction of plastic food containers and beverage cups. Includes product design, labeling, and waste management measures.	Estimated to significantly reduce plastic litter. Quantitative MP reduction not specified.
EU Microplastic Restriction [162]	EU/2023	Restricts intentionally added microplastics in consumer and industrial products. Targets items such as artificial sports turf infill, cosmetics, cleaning products, fertilizers, and more.	Estimated to prevent 500 kt of MPs over 20 years.
UN Global Plastics Treaty (Draft) [14]	UN/Expected 2025	A legally binding international treaty under negotiation. Targets the full life cycle of plastics: production, design, use, and waste management. Aims to include caps on virgin plastic production and MP reduction targets.	Global MP reduction targets under negotiation. Expected to drive large‑scale reduction.

Another critical upstream policy has been the banning of plastic microbeads in consumer products [135]. Microbeads -- tiny plastic scrubbers used in exfoliants and toothpastes -- were identified as needless contributors to waterborne MPs and NPs [136]. The United States passed the Microbead-Free Waters Act of 2015, prohibiting the manufacture and sale of rinse-off cosmetics containing plastic microbeads [137]. Canada, the UK, the EU, Australia, and others followed with similar bans [138]. These actions effectively eliminated a point source of MP pollution and pushed industry toward biodegradable alternatives such as salt or groundnut shells [139]. In 2022, the EU expanded its scope by adopting a sweeping REACH restriction targeting all intentionally added MPs in products -- including microbeads in cosmetics, pellets in detergents, and other polymer fillers [140]. This policy, which phases in from 2025 onward, is projected by the European Chemicals Agency (ECHA) to prevent approximately 500,000 tons of MPs from entering the environment over 20 years [141]. UK monitoring found >90% fewer plastic micro-scrubbers in wastewater following its 2018 ban, underscoring that well-crafted, source-specific bans can deliver measurable impact (Table 6) [142].

**Table 6:** Policy effectiveness assessment with gaps analysis Effectiveness data represent the best available estimates from policy evaluations and may vary by region and implementation rigor. "Implementation gaps" reflect current limitations identified in peer-reviewed assessments. MPs: microplastics.

Policy Instrument	Jurisdiction/Year	Scope	Quantitative Effectiveness	Implementation Gaps	Source
Microbead‑Free Waters Act	US/2015	Bans plastic microbeads in rinse‑off cosmetics	99% reduction in microbeads in targeted products; 95% manufacturer compliance	Does not address production caps for virgin plastics; no restrictions on industrial MP use	McDevitt et al., 2017 [143]
EU Single‑Use Plastics Directive	EU/2019	Bans plastic straws, cutlery, cotton swabs, polystyrene containers; mandates consumption reduction	80% compliance in member states; 90% reduction in targeted littered items on beaches	Enforcement varies by member state; limited impact on non‑SUP sources	Kiessling et al., 2023 [134]; Street et al., 2025 [5]
EU REACH MP Restriction	EU/2023 (phasing 2025+)	Restricts intentionally added MPs in all products	Projected 500,000 tons MP prevention over 20 years	Temporary exemptions for certain industries; does not address unintentional MP release	Catone et al., 2024 [140]
UN Global Plastics Treaty (Draft)	Global/Expected 2025	Addresses full plastic life cycle; production caps under negotiation	Targets under negotiation; potential for 30-50% MP reduction if ambitious targets adopted	Treaty not yet finalized; enforcement mechanisms unclear; voluntary vs. binding commitments debated	Fletcher & Evans, 2025 [14]

Microbeads still represent a very visible but quantitatively small slice of the MP burden. Broader chemical regulation is also underway, particularly around hazardous plastic additives. Many countries regulate phthalates and bisphenol A (BPA) in products that contact food or children's items [144]. The EU and US restrict several phthalates (DEHP, DBP, BBP, DINP) in toys due to reproductive toxicity concerns. BPA was banned in baby bottles in the EU (2011) and the US (2012), a move that successfully reduced BPA levels in infants' urine [145]. Yet, this action sparked substitution with analogs like bisphenol S (BPS) and bisphenol F (BPF) -- chemicals that later proved to have similar endocrine-disrupting properties. A similar pattern unfolded with phthalates: bans on a few compounds led to rising use of substitutes like diisononyl phthalate (DINP) and diisodecyl phthalate (DIDP), which are now also under scrutiny. Toxicologically, many replacements are proving hazardous, prompting regulators to play catch-up with additional restrictions [146].

These additive-specific bans have demonstrated efficacy in context. After the EU restricted deca-BDE (a brominated flame retardant) in electronics, follow-up studies found significantly lower levels of this compound in European breast milk samples [147]. In Japan, the phase-out of certain PVC additives in food packaging corresponded with a measurable drop in those chemicals in packaged foods [148]. These cases show that targeted bans can reduce human exposure to harmful chemicals that may adhere to or be released from MPs. Still, such targeted measures only address parts of the problem. Banning a handful of phthalates in toys does little to stem emissions from other consumer and industrial plastics shedding MPs loaded with unregulated endocrine disruptors. Moreover, enforcement gaps and international inconsistencies can limit impact. Countries with strict additive bans may still import goods containing those same chemicals from regions with looser rules, effectively outsourcing the pollution. The EU's MP restriction also includes derogations (temporary exemptions) for certain industries and essential uses, meaning MP release will not be fully eliminated. As a result, MP exposure continues via other pathways [140].

Extended Producer Responsibility (EPR) schemes aim to address this by shifting end-of-life accountability to manufacturers [149]. Under EPR, producers are financially responsible for post-consumer waste management, incentivizing designs that are more recyclable and less toxic [150]. Companies may pay eco-modulated fees: higher for non-recyclable or hazardous plastics, lower for safer, more sustainable options [151]. EPR schemes are active in the EU (via the Packaging Waste Directive) and in several US states. These are reinforced by complementary measures such as deposit-return systems, which increase recycling rates for plastic bottles, and minimum recycled content mandates, which create demand for recycled materials [152].

On a global scale, momentum is building toward a coordinated response [153]. In 2022, the United Nations Environment Assembly (UNEA) committed to creating a legally binding Global Plastics Treaty by 2024, covering the full life cycle of plastics [154]. Ongoing negotiations (e.g., INC-5 in 2024) have focused on capping virgin plastic production and setting clear targets for MP reduction [155]. A recent analysis by the European Environment Agency concluded that voluntary industry actions and scattered bans have failed to prevent the continued rise in plastic pollution. It called for a unified global framework that includes upstream controls and phaseouts of hazardous additives [140].

Finally, public education and infrastructure improvements remain vital policy levers [156]. Anti-litter campaigns, stormwater capture upgrades, and funding for MP-focused research are being embedded in national strategies [157]. In 2023, the United States released a National Strategy to Prevent Plastic Pollution, with actions ranging from promoting reuse/refill business models to investing in materials innovation and removal technologies [158].

In conclusion, additive-specific and product-specific bans are necessary but insufficient [159]. They serve as valuable first steps, but MP pollution and its associated chemical risks require a multipronged regulatory strategy. This includes not only eliminating the worst actors but also promoting safer material innovation, strengthening global oversight, improving waste management, and, perhaps most critically, curbing unnecessary plastic production at the source. A comprehensive framework, such as the global treaty now in development, offers the best chance of addressing plastics' full life cycle and preventing today's solutions from becoming tomorrow's new hazards [160].

Limitations

This narrative review has several important limitations that should be considered when interpreting its findings.

Review Design and Search Strategy

This is a narrative rather than a systematic review. Although we conducted a targeted search of PubMed, Scopus, and Web of Science for articles and policy reports published between 2010 and 2025, the search was not exhaustive and did not follow a preregistered systematic review protocol. We restricted inclusion to English-language publications and a defined set of keywords, which may have excluded relevant studies in other languages, gray literature, and work indexed in other databases. Study selection and interpretation, therefore, reflect the author's judgment, and publication bias cannot be ruled out. Future systematic reviews with predefined protocols and formal risk-of-bias tools would strengthen evidence synthesis in this field.

Variable Data Quality and Heterogeneity

The underlying studies differ widely in design (laboratory, environmental monitoring, epidemiology), sampling strategies, particle size cutoffs, polymer identification methods, exposure metrics, and outcome definitions. This heterogeneity, together with uneven reporting of quality-control procedures, precluded quantitative meta-analysis and limited direct comparison of effect sizes across studies. Our synthesis is therefore descriptive and thematic rather than pooled, and readers should interpret cross-study contrasts cautiously.

Measurement and Detection Constraints

Many environmental and biomonitoring studies detect particles only down to approximately 300 µm, and most cannot reliably quantify NPs. As a result, reported MP concentrations likely underestimate the true particle burden, particularly at the nano-scale. Newer analytical approaches (for example, pyrolysis-GC/MS (gas chromatography/mass spectrometry) and advanced Raman or FTIR (Fourier-transform infrared) methods) are beginning to close this gap, but comprehensive nano-scale datasets remain scarce. Quantitative estimates in this review should be viewed as order-of-magnitude indicators rather than precise inventories.

Causality Versus Correlation in Health Outcomes

Throughout this review, associations between MP exposure and health outcomes are largely correlational and observational. Many studies have small sample sizes, limited follow-up, and incomplete control for confounding factors, including co-exposures to other pollutants. Consequently, current evidence supports plausibility and correlation rather than definitive causal inference. Definitive causal pathways require longitudinal epidemiological studies, mechanistic toxicology research, and careful control of confounding variables. We have aimed to distinguish clearly between established associations and hypothesized mechanisms, but readers should apply appropriate caution when interpreting health implications.

Geographic, Temporal, and Life-Cycle Gaps

The literature is heavily weighted toward North America, Europe, and East Asia, with relatively few data from Africa, South America, and small island and polar regions. Temporal coverage is also uneven, with most high-quality monitoring and biomonitoring studies appearing after 2015. In addition, LCA data for emerging bioplastics and alternative materials are limited; few cradle-to-grave studies quantify MP release, real-world degradation kinetics, or toxicological profiles of breakdown products. These gaps constrain the generalizability of some conclusions and highlight priorities for future research.

Despite these limitations, this review provides an integrated overview of historical plastic production, environmental distribution, human exposure, and policy responses, while identifying areas where more systematic, high-quality evidence is needed.

Future research directions

Addressing the limitations identified above and advancing the field requires coordinated efforts across multiple research domains.

In analytical method development, priority should be given to establishing standardized protocols for NP detection and quantification below 1 μm with validated inter-laboratory reproducibility. The field would benefit substantially from harmonized reporting units and size classifications across studies, with a recommendation that researchers report particles per kg/L alongside mandatory size distribution data. Improved polymer identification techniques with lower detection limits (below 10 μm for routine analysis and below 1 μm for specialized applications) are needed, as is the development of field-deployable, real-time MP/NP sensors for environmental monitoring programs.

Health impact research represents perhaps the most critical knowledge gap. Longitudinal epidemiological cohorts tracking MP exposure biomarkers and health outcomes over 10 or more years are essential to move beyond cross-sectional associations. Mechanistic toxicology studies should focus on elucidating cellular and molecular pathways of MP/NP toxicity, while dose-response characterization is needed for priority polymer types, including polyethylene, polypropylene, polystyrene, and PET, as well as for common additives such as phthalates, BPA, and PFAS substances. Investigation of NP translocation across biological barriers, including the blood-brain barrier, placental barrier, and gut epithelium, deserves particular attention, given preliminary evidence of systemic distribution.

Environmental fate and transport research would benefit from global monitoring networks providing spatially and temporally resolved data using standardized methods. Advanced modeling of atmospheric MP transport and deposition patterns is needed, along with quantification of terrestrial-to-aquatic MP fluxes via runoff, wastewater discharge, and atmospheric deposition. Assessment of MP persistence and degradation kinetics across environmental compartments under realistic field conditions, rather than accelerated laboratory simulations, remains a significant gap.

Policy and intervention research should include rigorous evaluation of policy effectiveness using before-and-after controlled designs wherever possible. Economic analyses of extended producer responsibility schemes and plastic taxation would inform evidence-based policymaking, as would systematic assessment of technological solutions, including washing machine microfiber filters, tire wear capture systems, and improved waste management infrastructure. Cross-national policy comparisons could identify best practices and transferable approaches for different regulatory contexts.

Finally, green chemistry innovation requires the continued development of truly biodegradable polymers with validated marine and soil degradation under realistic environmental conditions rather than idealized laboratory settings. LCAs should incorporate MP generation potential as a standard metric, and toxicological profiling of biodegradation intermediates and end products is needed to ensure that solutions do not create new problems. Scalability and techno-economic analyses enabling bioplastic cost parity with conventional plastics would accelerate market adoption.

These research priorities require sustained funding, international coordination, and commitment to open data sharing to effectively address the global MP crisis.

## Conclusions

Since the invention of Bakelite in 1907, annual plastic production climbed from about 2 million metric tons in 1950 to more than 450 million metric tons in 2018, with an estimated 8.3 billion metric tons of virgin plastic produced in total within that timeframe. As of 2015, about 79% of that plastic waste accumulated in landfills or the environment, only 9% was recycled, and 12% was incinerated. Mismanaged coastal waste alone sent an estimated 4.8 to 12.7 million metric tons of plastic into the oceans in 2010, and current trends suggest that plastic leakage could rise by about 50% by 2040 without stronger policies. These figures define the scale of the MP reservoir that now permeates ecosystems and, increasingly, human tissues.

Early human data add a health signal to this mass balance. MPs and NPs have been detected in blood, lung tissue, placenta, brain tissue, and atherosclerotic plaques. Observational studies also link MP exposure to endocrine disruption and impaired sperm quality, although effect estimates vary and mechanisms remain uncertain. Together, these findings support biological plausibility and raise concern, even though they do not yet prove causality.

Two quantitative take-home messages follow from this synthesis. First, if current patterns continue, most of the 8.3 billion metric tons of plastic that have already been produced, and the additional billions projected this century, will remain as long-lived macro- and microplastic stocks unless we change production and waste trajectories. Second, targeted policies already show measurable potential: the EU’s new restriction on intentionally added MPs is expected to prevent about 500,000 metric tons of MP emissions over 20 years, and OECD (Organisation for Economic Co-operation and Development) modeling suggests that a high-stringency downstream package alone could reduce plastic leakage into the environment by roughly one-third compared with 2020 levels, while comprehensive life-cycle policies could cut leakage by more than 90% by 2040. These numbers illustrate both the magnitude of the problem and the scale of prevention that coordinated interventions can deliver.

In light of these findings, three priorities emerge. First, we need upstream action to slow the growth of the plastic stock: caps or reduction targets for primary plastic production, especially for short-lived applications, and substitution away from high-shedding polymers and additive packages of concern. Second, we need midstream design and technology measures that cut MP generation during use, including low-shed textiles, tire and road innovations, and filtration or capture systems that can realistically operate at scale. Third, we need downstream infrastructure and governance that close leakage pathways - universal waste collection, improved landfills, safe incineration where appropriate, and rigorous control of pellet and recycling losses - alongside longitudinal epidemiologic and mechanistic studies that clarify health risks and inform exposure limits.

MPs and NPs represent the durable footprint of a linear “take-make-dispose” materials system. Because plastics persist for decades to centuries, every delay in upstream and midstream intervention adds to a burden that waste management and remediation alone cannot erase. The evidence summarized here indicates that aligning production policy, product design, waste management, and health research offers a plausible path to flatten MP contamination curves and to reduce plausible cardiovascular, endocrine, and reproductive risks, even as causal pathways remain under study.
